# Enteric nervous system abnormalities are present in human necrotizing enterocolitis: potential neurotransplantation therapy

**DOI:** 10.1186/scrt387

**Published:** 2013-12-25

**Authors:** Yu Zhou, Jixin Yang, Daniel J Watkins, Laura A Boomer, Mika A Matthews, Yanwei Su, Gail E Besner

**Affiliations:** 1 The Center for Perinatal Research, The Research Institute at Nationwide Children’s Hospital, Columbus, OH 43205, USA; 2 Department of Pediatric Surgery, The Ohio State University College of Medicine, 700 Children’s Drive, Columbus, OH 43205, USA

## Abstract

**Introduction:**

Intestinal dysmotility following human necrotizing enterocolitis suggests that the enteric nervous system is injured during the disease. We examined human intestinal specimens to characterize the enteric nervous system injury that occurs in necrotizing enterocolitis, and then used an animal model of experimental necrotizing enterocolitis to determine whether transplantation of neural stem cells can protect the enteric nervous system from injury.

**Methods:**

Human intestinal specimens resected from patients with necrotizing enterocolitis (*n* = 18), from control patients with bowel atresia (*n* = 8), and from necrotizing enterocolitis and control patients undergoing stoma closure several months later (*n* = 14 and *n* = 6 respectively) were subjected to histologic examination, immunohistochemistry, and real-time reverse-transcription polymerase chain reaction to examine the myenteric plexus structure and neurotransmitter expression. In addition, experimental necrotizing enterocolitis was induced in newborn rat pups and neurotransplantation was performed by administration of fluorescently labeled neural stem cells, with subsequent visualization of transplanted cells and determination of intestinal integrity and intestinal motility.

**Results:**

There was significant enteric nervous system damage with increased enteric nervous system apoptosis, and decreased neuronal nitric oxide synthase expression in myenteric ganglia from human intestine resected for necrotizing enterocolitis compared with control intestine. Structural and functional abnormalities persisted months later at the time of stoma closure. Similar abnormalities were identified in rat pups exposed to experimental necrotizing enterocolitis. Pups receiving neural stem cell transplantation had improved enteric nervous system and intestinal integrity, differentiation of transplanted neural stem cells into functional neurons, significantly improved intestinal transit, and significantly decreased mortality compared with control pups.

**Conclusions:**

Significant injury to the enteric nervous system occurs in both human and experimental necrotizing enterocolitis. Neural stem cell transplantation may represent a novel future therapy for patients with necrotizing enterocolitis.

## Introduction

Necrotizing enterocolitis (NEC) is the most common gastrointestinal emergency in neonates and the leading cause of death in premature babies [1]. Current overall mortality rates are ~30%, with higher rates for very low birth weight babies. This high morbidity and mortality represent a significant medical problem and a costly burden to society.

Although the pathogenesis of NEC is not completely understood, prematurity is considered the most important risk factor for the development of the disease. In addition, exclusive formula feeding is associated with an increased incidence of NEC, while promotion of breast milk feeding may decrease the incidence of NEC [2-4]. The enteric nervous system (ENS), located in the wall of the intestine, is the largest and most complex division of the peripheral nervous system. The newborn ENS is not fully developed at birth and undergoes neuroplasticity during early postnatal stages [5]. The content of neurotrophic factors and cytokines in human breast milk contribute to the postnatal development of the ENS [6]. An immature ENS has been observed in premature infants, represented as decreased organized intestinal motility. It is thought that this intrinsic ENS immaturity may contribute to the development of NEC [7]. Intestinal motility is mainly regulated by neurotransmitters produced by myenteric neurons. Neuronal nitric oxide synthase (nNOS)-producing neurons and choline acetyl transferase (chAT)-producing neurons are involved in the regulation of intestinal motility, and nNOS/chAT misbalance has been reported in certain intestinal motility disorders [8]. Loss of enteric neurons in the submucosa has been demonstrated in patients with NEC, and some reports have shown NEC-induced myenteric plexus alterations [9,10]. In addition, post-NEC complications such as intestinal dysmotility, stricture, and recurrent abdominal distention have been widely reported [11,12]. The intestinal dysfunction that is present after successful medical or surgical management of NEC suggests that the compromised ENS is not fully recovered from the acute intestinal insult.

Similar to the central nervous system, neurons in the ENS have restricted potential for regeneration after injury. Limited neurogenesis may lead to a failure to reverse the neuronal cell loss that occurs during NEC, leading to compromised intestinal function long after recovery from the acute disease. Transplantation of intestinal enteric neurons to restore the neuronal cell loss associated with NEC may represent a future therapeutic option.

In the current study we have documented ENS abnormalities in patients with NEC. Furthermore, we have administered neural stem cells (NSCs) to rat pups with experimental NEC, documented engraftment of the transplanted NSCs in injured intestine, and confirmed improved ENS integrity and intestinal motility post transplantation.

## Materials and methods

### Human intestinal tissue collection

Approval for the use of human intestinal tissues was obtained from the Institutional Review Board of Nationwide Children’s Hospital, Columbus, OH, USA (Protocol #06-00267). This protocol met the guidelines for waiver of informed consent. Intestinal tissue specimens were obtained from 18 neonates with acute NEC (gestational age range 24 to 37 weeks, mean gestational age 32.1 ± 2.7 weeks, age range 4 to 21 days; mean age 10.7 ± 5.5 days) and eight control patients (gestational age range 28 to 36 weeks, mean gestational age 35.9 ± 1.4 weeks, age range 1 to 6 days; mean age 1.7 ± 1.4 days) with small bowel atresia. All patients chosen for examination underwent initial bowel resection with ostomy creation followed by subsequent stoma closure 2 to 3 months later. Intestinal samples were obtained from the antimesenteric border of the intestines for consistency, given that the ENS distribution in the intestine is variable [13]. Intestinal samples were subjected to RNA extraction, and to hematoxylin and eosin staining and immunohistochemistry.

### Neural stem cell culture

Enteric NSCs were generated using a modification of a previously described method [14]. Time mated c57BL/6-Tg (pan-EGFP) mice (Jackson Laboratory, Bar Harbor, MA, USA) were sacrificed and intestines from 12.5-day post‒coitum embryos were dissected into Dulbecco’s modified Eagle’s medium/Ham’s nutrient mixture F12 (DMEM/F12; Invitrogen, Carlsbad, CA, USA). Intestines were dissociated in 50 μg/ml dispase and 50 μg/ml collagenase (Worthington Biochemical, Freehold, NJ, USA) for 60 minutes at 37°C. Intestines were triturated and filtered through 40 μm cell strainers to obtain single cell suspensions. Cells were cultured in 35 mm Petri dishes in NSC culture medium consisting of DMEM/F12 containing 100 U/ml penicillin and 100 μg/ml streptomycin (Invitrogen), supplemented with 2 mmol/l l-glutamine (Invitrogen), 7.5% (v/v) chick embryo extract (Gemini Bio-products, West Sacramento, CA, USA), 1% (v/v) N_2_ medium supplement (Sigma‒Aldrich, St Louis, MO, USA), 20 ng/ml mouse basic fibroblast growth factor, and 20 ng/ml mouse epidermal growth factor (Sigma‒Aldrich). NSCs grew as free-floating cellular aggregates known as neurosphere-like bodies.

### Animal model of experimental necrotizing enterocolitis

All animal procedures were approved by the Institutional Animal Care and Use Committee of the Research Institute at Nationwide Children’s Hospital (Protocol #04203AR). Experimental NEC was induced as we have described previously [15,16]. Rat pups were delivered on day 21 of gestation by Cesarean section from timed pregnant rats (Harlan Sprague–Dawley, Indianapolis, IN, USA). Newborn rat pups in the experimental NEC group were maintained in an incubator at 37°C and gavage fed with hypertonic formula containing 15 g Similac 60/40 (Ross Pediatrics, Columbus, OH, USA) in 75 ml Esbilac (Pet-Ag, New Hampshire, IL, USA), a diet that provided 836.8 kJ/kg daily. Pups were exposed to hypoxia with 100% nitrogen for 1 minute followed by hypothermia at 4°C for 10 minutes twice daily beginning 60 minutes after birth for 3 days, with intragastric feeding of lipopolysaccharide (2 mg/kg; Sigma‒Aldrich) 8 hours after birth.

### Animal model of recovery from necrotizing enterocolitis

Animals that survived the exposure to experimental NEC described above were recovered by discontinuing exposure to hypoxia/hypothermia on day 3 of life, and then switching pups to normal formula feeding for an additional 4 days to mimic recovery from NEC. On day 3, an intraperitoneal (i.p.) injection of NSCs was performed as described by Martucciello and colleagues [17]. Experimental pups received a single i.p. injection of NSC (50,000 cells in 50 μl DMEM/F12) into the right lower quadrant, while control animals received the same amount of medium carrier intraperitoneally. Normal control pups, designated the breast milk group, were breast fed using surrogate mothers and were not exposed to stress.

### Immunohistochemistry and confocal microscopy

Tissue sections (4 μm thickness) from 4% paraformaldehyde-fixed paraffin-embedded human or rat intestine were subjected to immunohistochemistry with specific primary antibodies: mouse anti-human neuronal protein HuC/D (10 μg/ml), rabbit anti-nNOS (2 μg/ml), chicken anti-green fluorescence protein (GFP; 1 μg/ml) (all from Invitrogen); chicken anti-glial fibrillary acid protein (GFAP; 1:1,000), rabbit anti-protein gene product 9.5 (1:500), rabbit anti-peripherin (1:100) (all from Millipore, Billerica, MA, USA); and rabbit anti-cleaved caspase 3 (1:500; Cell Signaling, Danvers, MA, USA). After being washed in phosphate-buffered saline, sections were incubated with fluorophore-conjugated goat anti-mouse IgG (Alexa 647; Molecular Probes, Eugene, OR, USA), anti-rabbit IgG (Alexa 488; Molecular Probes) or anti-chicken Igγ (Cy3; Jackson ImmunoResearch Laboratories, West Grove, PA, USA) at room temperature. Sections were counterstained with 4′,6-diamidino-2-phenylindole. For immunocytochemistry of NSCs, free-floating neurosphere-like bodies were seeded in poly-d-lysine/laminin-coated eight-well chamber slides (BD Bioscience, Bedford, MA, USA) and cultured for an additional 12 hours. To detect nestin expression, neurospheres were incubated with mouse monoclonal antibody Rat-401 (1:100; Developmental Studies Hybridoma Bank of the University of Iowa, Iowa City, IA, USA) overnight at 4°C and then recognized by fluorophore-conjugated goat anti-mouse IgG (Cy3; Jackson ImmunoResearch Laboratories). For NSC differentiation studies, neurospheres were grown in differentiation medium (DMEM/F12 + 10% fetal bovine serum) for 4 days. Differentiated cells were recognized by monoclonal rabbit anti-neuronal class III β-tubulin (Tuj1, 1 μg/ml; Covence, Princeton, NJ, USA) and polyclonal chicken anti-GFAP (1:1,000) respectively. Fluorescence images were captured by confocal microscopy (LSM 710; Carl Zeiss, Thornwood, NY, USA). Images were analyzed using image analysis software (Image Pro Plus; Media Cybernetics, Silver Springs, MD, USA).

### Morphology of myenteric plexuses

Cross-sections from human intestinal specimens were used to identify enteric ganglia in myenteric plexuses located between the intestinal longitudinal and circular muscular layers. Intestinal tissue strips were dissected from the anti-mesenteric border of the intestines, and 10 serial sections with a thickness of 4 μm each were examined in order to reduce the possibility of biased observations. Human myenteric plexuses were identified by groups of HuC/D immunoreactive neuronal somata surrounded by glial cells. Total myenteric ganglion numbers in each section were recorded. Neurons were recognized by HuC/D-immunoreactivity, which was displayed as an artificial gray color, and neurons were quantified as the mean neuronal number per ganglion. nNOS-expressing functional neurons immunoreactive for nNOS were also counted, and the ratio of nNOS-expressing neurons to total HuC/D immunoreactive neurons per ganglia was calculated. The appearance of glial cells in the myenteric plexus, as displayed by GFAP immunoreactivity, showed very small nuclei with elongated and interconnected processes in the myenteric plexus, making it very difficult to count the glial cells directly. Since myenteric ganglia mainly consist of neurons and supportive glial cells, total glial cell numbers in each ganglion were extrapolated by subtracting the number of neurons from the total cell numbers as recognized with 4′,6-diamidino-2-phenylindole staining. Lastly, the myenteric neuronal cell area (HuC/D^+^-stained area) and the myenteric ganglion area were measured in blinded samples using Image Pro Plus software (Media Cybernetics), and the ratio of the areas was calculated [18].

### Real-time reverse-transcription polymerase chain reaction

Longitudinal muscle–myenteric plexus (LMMP) strips consisting of the myenteric plexus and the innervated longitudinal muscles were dissected from fresh human intestines. Total RNA was reverse-transcribed with random hexamers using a first-strand cDNA synthesis kit (Invitrogen-Gibco). Real-time reverse-transcription polymerase chain reaction (RT-PCR) was carried out using a SYBR Green RT-PCR kit (Applied Biosystems, Branchburge, NJ, USA) and an ABI Prism 770 Sequence Detection System (Applied Biosystems). Human nNOS and chAT were amplified using the following primers: nNOS sense, 5′-GGCCCATATTAATCCCTCGT-3′ and nNOS anti-sense, 5′-ACATGAGGGCTCTGCTCACT-3′; chAT sense, 5′-CCACTCCATTCCCACTGACT-3′ and chAT anti-sense, 5′-GAGACGGCGGAAATTAATGA-3′. Amplification of the housekeeper gene (glyceraldehyde 3-phosphate dehydrogenase) cDNA was used as an internal control for quantification. Quantification was performed using Relative Quantification Software, version 1.01 (Applied Biosystems).

### Western blotting

LMMP strips of intestines from rat pups were isolated 4 days after NSC transplantation. Tissue lysates were subjected to SDS-PAGE and proteins were transferred to nitrocellulose membranes and subjected to immunoblot analysis. Peripherin, GFAP, and nNOS were detected using primary antibodies followed by horseradish peroxidase-conjugated secondary antibodies. Blots were developed with the ECL Plus system (Amersham Biosciences, Piscataway, NJ, USA) using Hyperfilm (Amersham Biosciences) for exposure. The intensity of immunoreactive bands on western blots was quantified using the Scion Image Program (Scion Corporation, Frederick, MD, USA) and expressed as the mean ± standard error of the mean.

### Intestinal motility assay

Four days after NSC transplantation, rat pups were made nil *per os* for 4 hours followed by administration of 0.2 ml methylene blue-labeled 10% dextrose solution (Sigma-Aldrich) delivered by duodenal gavage. Animals were euthanized 5 minutes after introduction of the dye. Intestinal transit was measured from the pylorus to the most distal point of migration of the blue dye. The total length of the small intestine was measured and the ratio of dye migration distance/total intestinal length was calculated and transit times were expressed as a percentage of the total intestinal length [19].

### Statistical analyses

All data are presented as the mean ± standard error of the mean. Statistical analyses were performed using the Student *t* test or one-way analysis of variance (SigmaPlot 11.0; Systat Software, Inc. San Jose, CA, USA). Absolute *P* values are shown, with *P* <0.05.

## Results

### Enteric nervous system is injured in human necrotizing enterocolitis

**Figure 1:**
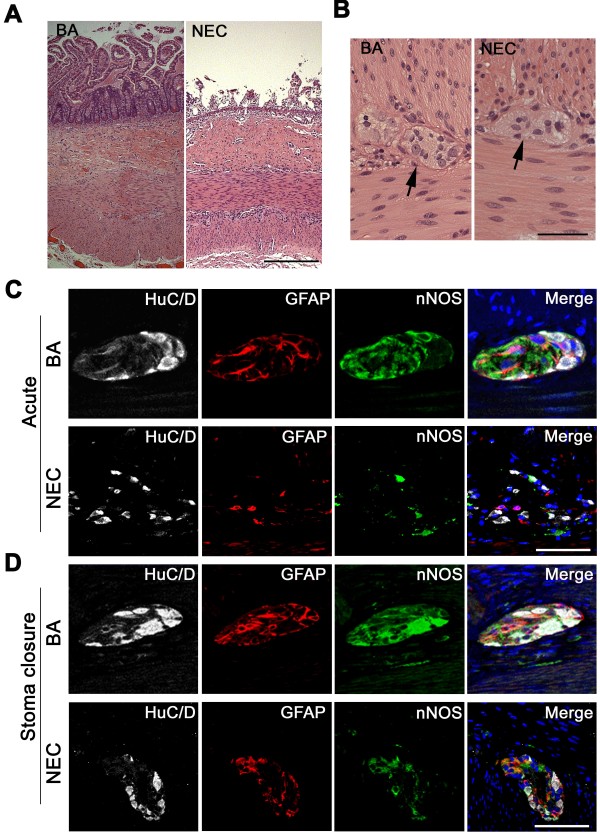
**Structural and functional abnormities exist in myenteric plexus ganglia of necrotizing enterocolitis patients. (A)** Representative hematoxylin-and-eosin-stained sections from human small intestine resected for small bowel atresia (BA) in a newborn 33-week-gestation infant compared with an 11-day-old 31-week-gestation baby with necrotizing enterocolitis (NEC). The control intestine shows a normal appearance of the intestine whereas epithelial sloughing was present in the intestine resected for NEC. **(B)** High-magnification images of myenteric ganglia (arrows) from a BA patient and a NEC patient (arrows). Ganglion cells are present in the myenteric plexus of intestinal sections in both images. **(C)**, **(D)** Myenteric ganglia were subjected to immunohistochemistry using antibodies to HuC/D (gray staining of total neurons), glial fibrillary acid protein (GFAP; red staining of glial cells), and neuronal nitric oxide synthase (nNOS; green staining of functional neurons producing nNOS). 4′,6-Diamidino-2-phenylindole was used to counterstain nuclei. **(C)** Bowel resected for acute disease. **(D)** Bowel resected during stoma closure 2 to 3 months later. Scale bar: **(A)** 10 μm; **(B)**, **(C)**, **(D)** 50 μm.

We compared human intestinal specimens from patients undergoing initial bowel resection for small bowel atresia or for NEC, as well as stoma closure specimens several months later. A review of hospital pathology reports revealed no mention of pathological changes in the ENS of these patients. We began by assessing histological changes in hematoxylin-and-eosin-stained tissue sections. Compared with control intestine, acute NEC was associated with severe epithelial damage (Figure 1A). Ganglion cells were present in the myenteric plexus of NEC patients (Figure 1B). We then performed immunohistochemistry using antibodies against HuC/D (a general neuronal marker to identify all neurons), GFAP (to identify glial cells), and nNOS (to identify nNOS-producing neurons). Whereas myenteric ganglia from bowel atresia specimens contained densely populated HuC/D-labeled neurons packed together in a characteristically tight fashion, ganglia from acute NEC specimens contained loosely packed HuC/D-labeled neurons with varying degrees of interneuronal spaces and considerable variation in cell body appearance (Figure 1C). Significantly decreased immunostaining for HuC/D, GFAP and nNOS was present in the myenteric ganglia of acute NEC specimens compared with bowel atresia specimens (Figure 1C). GFAP-positive glial cells and nNOS-expressing cells remained low in the ganglia of stoma specimens from patients who underwent initial bowel resection for NEC compared with stoma specimens from patients who underwent initial surgery for bowel atresia (Figure 1D).

**Figure 2:**
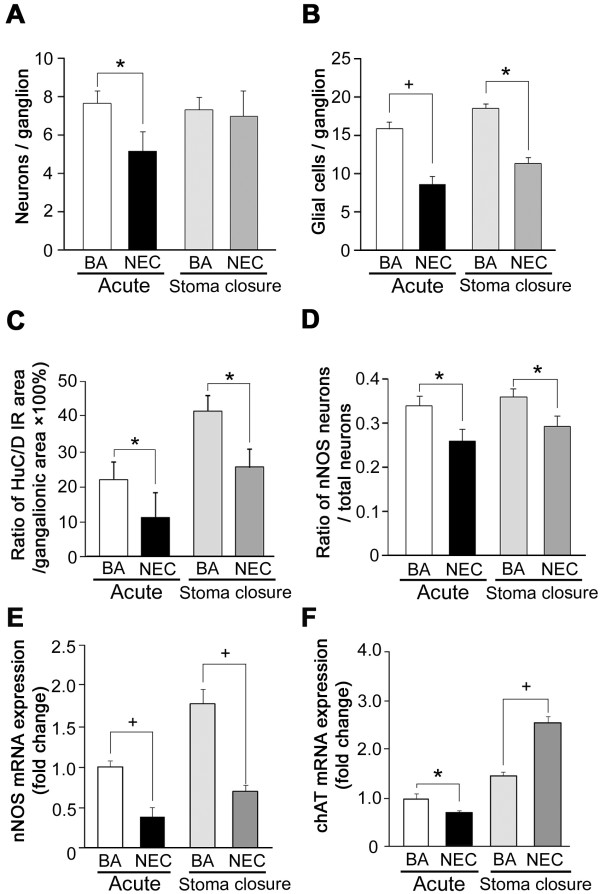
**Neuronal and glial cell loss occurs in myenteric plexus ganglia of necrotizing enterocolitis patients.** Intestinal specimens were examined from patients with acute necrotizing enterocolitis (NEC; *n* = 18), from age-matched patients with small bowel atresia (BA; *n* = 8), from NEC patients undergoing stoma closure (*n* = 14), and from BA patients undergoing stoma closure (*n* = 6). **(A)** Neuronal cell numbers in the myenteric ganglia were counted and neurons per ganglion presented as mean ± standard error of the mean. **(B)** The number of glial cells per ganglion was extrapolated by subtracting the number of HuC/D-positive neurons from the total numbers of with 4′,6-diamidino-2-phenylindole-labeled nuclei. **(C)** The ratio of the cytoplasmic area of HuC/D immunoreactive (IR) neurons to myenteric ganglionic area was calculated. **(D)** Neuronal nitric oxide synthase (nNOS) and HuC/D double-labeled cells in myenteric plexuses were counted and the ratio of nNOS-expressing neurons to total HuC/D stained neurons was calculated. **(E)** mRNA expression of nNOS as determined by real-time reverse-transcription polymerase chain reaction (RT-PCR). **(F)** mRNA expression of choline acetyl transferase (chAT) as determined by real-time RT-PCR. *P <0.05; ^+^*P* <0.01.

Quantification of the numbers of cells per ganglion confirmed that the number of HuC/D-positive total neurons per ganglia was significantly reduced in specimens from patients with acute NEC compared with bowel atresia (5.19 ± 1.20 vs. 7.62 ± 0.61, *P* <0.05) (Figure 2A). The number of glial cells per ganglia was also significantly reduced in specimens from patients with acute NEC compared with bowel atresia (8.41 ± 1.12 vs. 15.8 ± 0.82, *P* <0.05), and these glial cell numbers remained low months later at the time of stoma closure (11.2 ± 0.73 vs. 18.4 ± 0.67, *P* <0.05) (Figure 2B). Although both neuronal and glial cell loss was found in the myenteric plexuses of NEC patients, there was no reduction in the overall size of the myenteric plexuses in NEC patients, either during acute NEC or at the time of stoma closure (during acute disease: NEC 27,197 ± 7,125 μm^2^ vs. bowel atresia 29,019 ± 12,310 μm^2^, *P* >0.05; at stoma closure: NEC 29,227 ± 9,145 μm^2^ vs. bowel atresia 30,115 ± 7,125 μm^2^, *P* >0.05). However, the ratio of the cytoplasmic area of HuC/D immunoreactive neurons to myenteric ganglion area or neural packing density was significantly decreased in NEC patients compared with patients with bowel atresia (Figure 2C). In addition, the ratio of nNOS-expressing neurons to total HuC/D immunoreactive neurons was significantly decreased in the myenteric ganglia from NEC patients compared with bowel atresia patients both during the acute episode (0.26 ± 0.04 vs. 0.34 ± 0.03) and later during stoma closure (0.29 ± 0.03 vs. 0.36 ± 0.02) (Figure 2D).

Since nNOS and chAT are important regulators of intestinal motility, we next investigated nNOS and chAT mRNA expression in our intestinal samples. Since nNOS-expressing neurons are abundant in the myenteric plexus compared with the submucosal plexus [20], we intentionally removed the circular muscular layer and its tightly attached submucosal plexus in order to prepare LMMP strips for RT-PCR analysis of the myenteric plexus alone in these experiments. Real-time RT-PCR confirmed that acute NEC was associated with significantly decreased expression of both nNOS and chAT (Figure 2E, F). Whereas nNOS expression remained low, chAT expression increased at the time of stoma closure. The disturbed expression of nNOS and chAT in the intestine during and after NEC suggests that ENS functions are disrupted even during the recovery phase.

**Figure 3:**
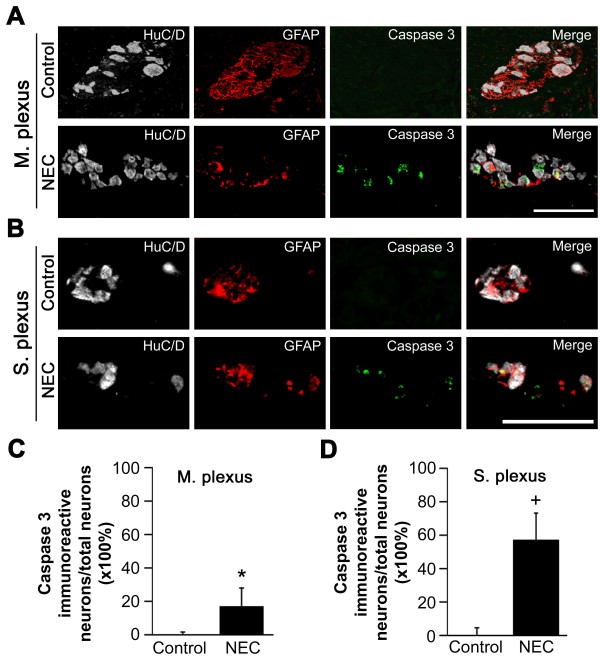
**Increased apoptosis is present in the myenteric plexus ganglia of necrotizing enterocolitis patients. (A)**, **(B)** Myenteric ganglia and submucosal ganglia respectively were subjected to immunohistochemistry using antibodies to HuC/D (neurons), glial fibrillary acid protein (GFAP; glial cells), and cleaved caspase 3 (apoptotic cells). Scale bar: 50 μm. **(C)**, **(D)** Cleaved caspase-3-positive neurons were counted and expressed as the percentage of total HuC/D stained neurons in ganglia from the myenteric plexus (M. plexus) and the submucosal plexus (S. plexus), respectively. Values represent mean ± standard error of the mean, with data generated from the intestines from 11 patients with acute necrotizing enterocolitis (NEC) and six patients with small bowel atresia (BA). *P < 0.05 versus control, ^+^*P* < 0.01 versus control.

To determine whether the neuronal cell loss found in NEC was due to apoptosis, we assessed neuronal apoptosis by cleaved caspase-3 immunohistochemistry. Associated with the decreased numbers of total neurons and glial cells, we found increased numbers of caspase 3-positive cells per ganglia in the myenteric plexus and submucosal plexus of intestine resected for NEC compared with control intestine (Figure 3A, B). Quantification revealed that the numbers of cleaved caspase-3-positive cells per ganglia were significantly increased in the myenteric plexus of specimens from acute NEC patients compared with bowel atresia patients (17.2 ± 3.1% vs. 1.5 ± 0.87%, *P* <0.05) (Figure 3C). Significantly increased neuronal apoptosis was also present in the submucosal plexus of specimens from acute NEC patients compared with bowel atresia patients (58.7 ± 7.6 vs. 4.9 ± 2.7%, *P* <0.05) (Figure 3D).

### Generation of neurospheres from fetal intestine

**Figure 4:**
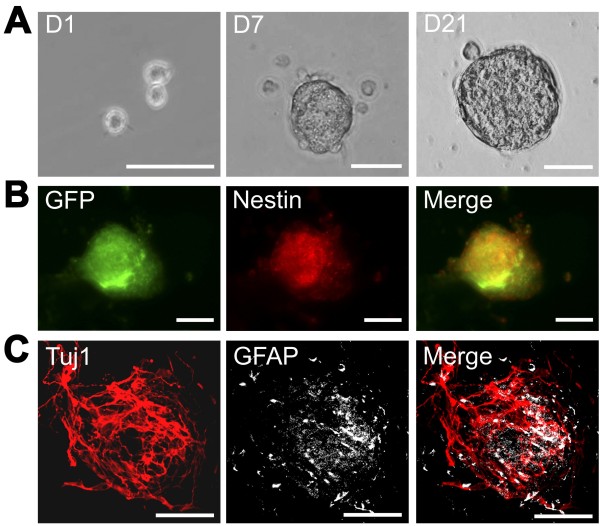
**Morphologic and immunocytochemical analysis of enteric neurospheres. (A)** Appearance of neurospheres generated from neural stem cells (NSCs) isolated from embryonic day 12.5 intestine of pan-EGFP mice grown for 1, 7 and 21 days in culture. **(B)** Appearance of neurospheres on day 21 of culture showing green fluorescent protein (GFP) labeling of all cells, immunostaining for nestin (specific marker of neural stem cells), and the merged images. **(C)** Neurospheres were induced to differentiate into neurons and glial cells after withdrawal of growth factors, and were grown in poly-d-lysin/laminin-coated slide chambers for 4 days. Differentiated neurons and glial cells were recognized by Tuj1 and glial fibrillary acid protein (GFAP) staining respectively. D1, day 1; D7, day 7; D21, day 21. Scale bars: 50 μm.

Previous studies have demonstrated that NSCs isolated from embryonic guts were able to proliferate in response to epidermal growth factor and basic fibroblast growth factor in serum-free culture conditions to generate clonal aggregates of undifferentiated neural precursor cells [21,22]. In agreement with the studies from other laboratories [23], separate clonogenic assays of NSCs from fetal or postnatal intestinal tissue were performed in our laboratory, and demonstrated a higher proliferation rate of fetal NSC colonies than their postnatal counterparts. Based on these findings, fetal intestine was harvested from pan-EGFP mice at embryonic day 12.5 and dissociated with enzymatic digestion. From days 1 to 21, colonies grew in size and formed neurospheres (Figure 4A). Nestin immunostaining confirmed the presence of significant numbers of neural precursor cells in the neurospheres and EGFP-labeled cells were easily visualized by the green florescence (Figure 4B). After withdrawal of growth factors, NSCs were induced to differentiate into neurons and glial cells that were recognized by Tuj1 and GFAP immunostaining respectively, confirming that enteric NSCs are the lineage precursors to neuronal and glial cells (Figure 4C).

### Enteric nervous system is injured in experimental necrotizing enterocolitis

**Figure 5:**
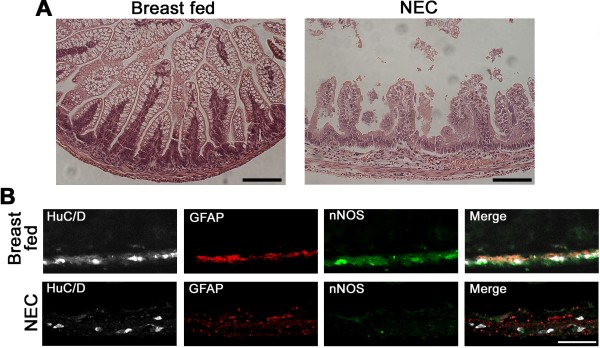
**Injury to the enteric nervous system occurs in experimental necrotizing enterocolitis. (A)** Representative hematoxylin-and-eosin-stained sections from a breast-fed rat pup with normal intestine, and a rat pup exposed to experimental necrotizing enterocolitis (NEC) for 3 days with epithelial sloughing and villous necrosis. **(B)** HuC/D, glial fibrillary acid protein (GFAP), and neuronal nitric oxide synthase (nNOS) immunohistochemistry, with merged images shown.

Similar to human NEC, intestinal sections from pups exposed to experimental NEC demonstrated epithelial cell sloughing and villous necrosis (Figure 5A). Again similar to our human NEC findings, pups subjected to experimental NEC had decreased total neuronal cells, glial cells, and nNOS-expressing cells in the injured intestines compared with breast-fed pups (Figure 5B).

### Neural stem cell transplantation preserves enteric nervous system integrity in experimental necrotizing enterocolitis

**Figure 6:**
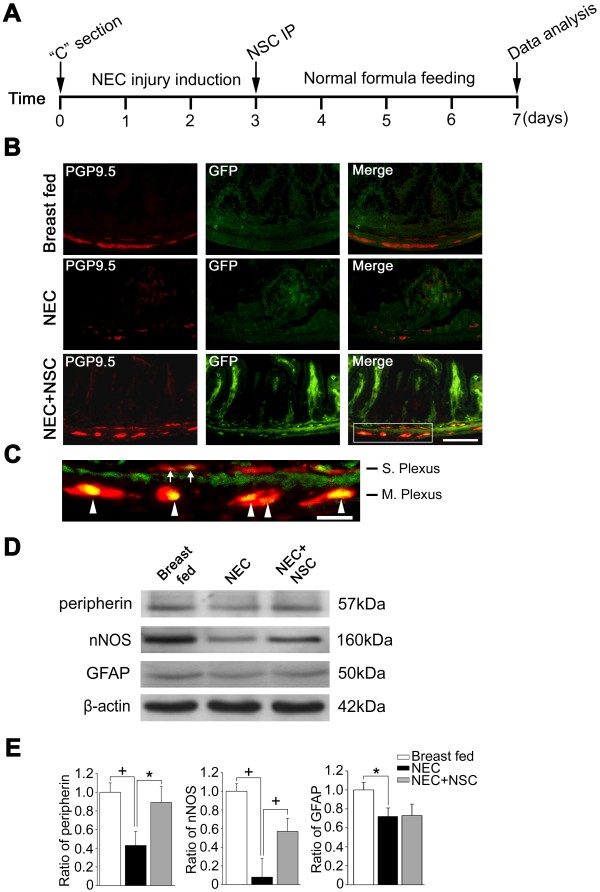
**Enteric neural stem cell transplantation protects the intestines in experimental necrotizing enterocolitis. (A)** Outline of the experimental protocol used. Rat pups were delivered by Cesarean section and then exposed to repeated episodes of hypoxia, hyperthermia, and hypertonic formula feeding for 3 days to induce experimental necrotizing enterocolitis (NEC). Pups then received intraperitoneal (IP) administration of green fluorescence protein (GFP)-labeled neural stem cells (NSCs) or carrier medium only, after which they were maintained on normal feeds with discontinuation of stress for an additional 4 days. **(B)** Representative images of intestinal sections from breast-fed rat pups, from pups exposed to NEC, and from pups exposed to NEC but treated with NSC transplantation. GFP-labeled transplanted cells were visualized in intestinal tissue sections by anti-GFP immunohistochemistry, and were further characterized by protein gene product 9.5 (PGP9.5) staining to identify mature neurons. **(C)** High magnification of the merged image inset (white box) demonstrating that transplanted NSCs have engrafted into the submucosal plexus (S. Plexus; arrows) and myenteric plexus (M. Plexus; arrowheads) of the intestine and have differentiated into PGP9.5-positive mature neurons (merged yellow staining). **(D)** Western blot analysis of intestinal longitudinal muscle–myenteric plexus strips showing protein expression of peripherin and neuronal nitric oxide synthase (nNOS). **(E)** Quantification of the intensity of immunoreactive bands: the band intensity ratios of peripherin to β-actin, nNOS to β-actin, and glial fibrillary acid protein (GFAP) to β-actin were calculated and are expressed as mean ± standard error of the mean, *n* = 4. *P <0.05; ^+^*P* <0.01. Scale bars: **(B)** 100 μm; **(C)** 25 μm.

We developed a novel model for recovery from NEC-induced intestinal dysmotility in which rat pups are exposed to stress (hypoxia/hypothermia/hypertonic feeds) for 3 days, and are then maintained on normal feeds without stress for an additional 4 days (Figure 6A). As opposed to the standard NEC model, which is useful for assessing intestinal pathology during acute NEC, this modified NEC model demonstrates changes in the intestines that are present during recovery from NEC. We delivered GFP-labeled enteric NSC via i.p. injection after the 3-day period of stress. To determine whether transplanted enteric NSCs home to the ENS and differentiate into neurons, we performed double immunofluorescence staining for GFP and for the pan-neuronal marker protein gene product 9.5. We found that engrafted GFP-positive cells were distributed in the muscularis, mucosa and villi of the recipient intestine (Figure 6B). Some transplanted cells were located in close proximity to the submucosal and myenteric plexuses of the host intestine (Figure 6C). A subset of the GFP-labeled engrafted cells were also positive for protein gene product 9.5, indicating differentiation of these NSCs into mature neurons post transplantation (Figure 6C). The engrafted NSCs did not stain positive for the glial cell marker GFAP (data not shown), indicating that the engrafted NSCs did not differentiate into glial cells after transplantation. In addition, western blot analysis of LMMP strips revealed decreased expression of the pan-neuronal marker peripherin, the glial cell marker GFAP, and nNOS in rat pups with NEC. However, NSC implantation led to a significant increase in the expression of peripherin and nNOS 4 days after transplantation (Figure 6D, E).

### Neural stem cell transplantation protects the intestines, promotes motility, and increases survival in experimental necrotizing enterocolitis

**Figure 7:**
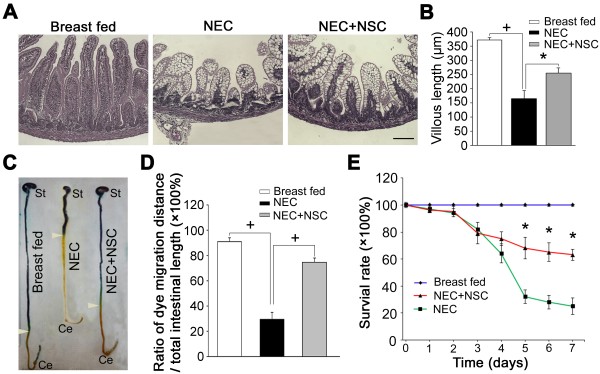
**Enteric neural stem cell transplantation restores intestinal integrity, promotes intestinal motility, and improves survival after experimental necrotizing enterocolitis. (A)** Representative hematoxylin-and-eosin-stained histologic images of ileal sections from pups exposed to breast feeding, experimental necrotizing enterocolitis (NEC), or experimental NEC treated with neural stem cell (NSC) transplantation. **(B)** Quantification of villous length, measured as the distance from the crypt neck to the villous tip using Image J software (NIH, Bethesda, MD, USA). Data presented as mean ± standard error of the mean (SEM), *n* = 6 animals in each group. *P <0.05; ^+^*P* <0.01. **(C)** Representative images of rat pups with methylene blue dye migration determined at day 7 after birth. White arrowheads indicate the most distal aspect of dye migration. St, stomach; Ce, cecum. **(D)** Effect of neurotransplantation on intestinal transit of methylene blue. Dye migration was measured as the ratio of migration distance/total intestinal length. Results expressed as mean ± SEM. Six to eight pups were used in each group. ^+^*P* <0.01. **(E)** Survival rate of rat pups after experimental NEC with or without NSC transplantation. *n* = 28, *P <0.05 for NSC transplantation group versus control medium group.

Intestine from animals exposed to experimental NEC that received NSC transplantation had significantly longer villous length compared with intestine from nontransplanted animals (Figure 7A, B). Although the villous structure was well preserved, vacuolization in enterocytes was found in some rat pups that received NSCs. This could be due to the response of the enterocytes to exposure to hypoxia, hyperthermia and administration of hypertonic feeds in the NEC animal model. In addition, host immune rejection of the transplanted mouse NSCs could also lead to cytoplasmic vacuolization. Rat pups exposed to experimental NEC had significantly decreased intestinal motility after 4 days of recovery from NEC, whereas animals exposed to NEC that received NSC transplantation had significantly improved intestinal transit (71.3 ± 8.8% vs. 31.6 ± 11.4% dye migration distance, *P* <0.05) (Figure 7C, D). NSC transplantation also significantly improved the survival rate of animals subjected to experimental NEC at 2, 3 and 4 days post transplantation (Figure 7E).

## Discussion

Although the pathophysiology of NEC remains elusive, immaturity of the intestinal tract has been implicated in its development. The ENS is comprised of neurons and supportive glial cells present in two major plexuses, the myenteric plexus and the submucosal plexus, that control the physiologic functions of the gastrointestinal tract [24]. A significant reduction in glial cells, associated with a gradual deterioration of intestinal neurons, has been identified in intestine resected for NEC [9,10]. In this study, we have confirmed that NEC is associated with neuronal and glial cell loss in the ENS. Importantly, the numbers of enteric glial cells remained low in the intestines from NEC patients even months after the acute episode when the patients underwent stoma closure. This suggests that intrinsic immaturity of the ENS, or dysfunctional glial cells, may predispose premature babies to NEC. The decrease in glial cells with NEC is surprising given that ischemia or inflammation is a powerful stimulus to glial cell proliferation or GFAP expression, such as that which occurs with 2,4,6-trinitrobenzene sulfonic acid-mediated inflammation of the gastrointestinal tract [25], or in inflammatory diseases of the gut such as ulcerative colitis [26]. Acute failure of enteric glia could be an upstream event in the cascade that precipitates the ischemic insult resulting in NEC. A previous study showed that ablation of enteric glial cells leads to fulminant and fatal intestinal inflammation, which is very consistent with the histopathologic changes seen in NEC [27]. While the mechanisms of neuronal cell loss have not been studied in patients with NEC, deprivation of neurotropic factors such as nerve growth factor and glial-derived neurotrophic factor that are normally provided by supportive glial cells may lead to activation of apoptotic pathways. In the current study, we demonstrated the presence of neuronal apoptosis via activation of a caspase-3-dependent pathway. Strong activated caspase-3 immunoreactivity was evident in the myenteric and submucosal ganglia of the intestines from NEC patients, with similar findings observed in the ENS of animals subjected to experimental NEC. Recent reports showed that GFAP-positive glia exert an anti-apoptotic effect on intestinal epithelial cells and preserve mucosal integrity by limiting epithelial damage and promoting epithelial reconstitution during inflammation [28,29]. Dysfunctional glia in the immature ENS of premature newborns, or lack of growth factors in formula feeding, may contribute to the impairment of the epithelial lining that leads to acute NEC.

Loss of specific neuronal subpopulations in the myenteric plexus has been reported during aging or disease, or in animals with specific genetic defects, indicating that neuronal subpopulations respond differently to injury or growth factor deprivation [30-32]. We found that patients with NEC have loosely packed myenteric neurons, with small neurons remaining in the myenteric ganglia. It is possible that the larger myenteric neurons were affected more dramatically, leading to loss of these cells. Preferential loss of large ganglion cells has also been reported by others [33,34], and large motor neurons appeared to be more vulnerable to some diseases or to be the first group to die during injury [35,36]. Further study of the vulnerability of some specific neuronal subpopulations such as motor neurons during NEC is required.

Among the subpopulations of neurons in the ENS, nNOS-immunoreactive neurons are more abundant in the myenteric plexus compared with the submucosal plexus [20]. The constitutive expression of nNOS in the gastrointestinal tract maintains the regulated production of low levels of nitric oxide, which acts as a predominant inhibitory neurotransmitter. Nitric oxide, mainly produced by enteric neurons and glial cells, causes relaxation of the intestinal smooth muscle in response to nerve stimulation, which is important in gut motility [37,38]. Nitric oxide signaling also regulates proliferation and differentiation of neural precursor cells [39,40]. Although changes in nNOS with NEC have not been reported, there is one report of decreased nNOS expression in a rat model of lipopolysaccharide-induced intestinal injury [41]. This is the first report to demonstrate that clinical NEC is associated with significantly decreased expression of nNOS not only during the acute event, but also months later. This decreased nNOS expression may explain the long-term intestinal dysmotility seen in NEC patients even after recovery from the acute event.

Current therapy for intestinal dysmotility is limited mainly to palliation, and new treatments for this debilitating condition are clearly needed. A promising intervention involves NSC transplantation. In efforts to develop stem cells therapies for ENS disorders, several sources of NSCs have been explored, including NSCs derived from totipotent embryonic stem cells, NSCs derived from the fetal or adult central nervous system, NSCs derived from fetal or adult intestine (enteric NSCs) and NSCs derived from non-neuronal sources (mesenchymal stem cells, amniotic fluid-derived stem cells) [42]. Enteric NSCs are isolated from the intestine and form neurospheres that contain nestin-positive NSCs capable of differentiating into neurons and glial cells when growth factors are withdrawn. Enteric NSCs, by virtue of their intestinal origin, may be more suitable for therapeutic purposes since they may respond better to intestinal-specific environmental cues upon transplantation [42,43]. Several reports have already described functional success of NSC transplantation for other diseases. NSCs transplanted either by direct injection into the intestinal wall or by i.p. administration led to the engraftment of NSCs in the myenteric plexus, and the generation of neurons in ganglionated small bowel and colon as well as aganglionic rectum [17]. Whereas direct intramural injection resulted in clumps of cells, i.p. injection resulted in diffuse engraftment throughout the gastrointestinal tract, representing a clinically viable route of administration, especially if generalized areas of intestine need to be treated. We have previously shown that i.p. administration of mesenchymal stem cells is an effective delivery route of stem cells in experimental NEC, as it avoids passive entrapment of transplanted cells in the pulmonary capillary bed [16]. Based on this, we used i.p. administration of NSCs in the current study, and documented engrafted cells in the submucosal and myenteric plexuses after transplantation. However, i.p. injection of NSCs to breast-fed normal rat pups did not result in engraftment of cells in the intestines (data not shown), suggesting that the cytokines and chemokines secreted by the injured intestine in experimental NEC are crucial in directing the mobilization, migration and homing of stem cells to the injured ENS. Others have shown that enteric NSCs isolated from embryonic intestine were committed to differentiate into neurons and glial cells [44]. Although the precise local factors responsible for transplanted NSC differentiation have not been identified, our results suggest that the gut environment provides a predominantly neurogenic drive for NSCs, with differentiated neurons but not glial cells identified in the recipient intestine post transplantation.

For future clinical neurotransplantation in human patients with NEC, collection of NSCs from fetal gut or from postnatal myenteric plexuses is not clinically practical. In a parallel study, we found that NSCs can be selectively induced from amniotic fluid-derived multipotent stem cells. Based on this, amniotic fluid could be collected at the time of delivery, amniotic fluid-derived stem cells cultured, and the cells induced into NSCs in the laboratory. The abundant availability of amniotic fluid meets the requirement for large numbers of stem cells for transplantation. NSC transplantation may represent a novel future potential therapeutic strategy for those premature newborns at highest risk of developing NEC, or those with early stages of the disease.

## Conclusion

In summary, our results confirm significant ENS damage during clinical and experimental NEC. Engraftment of enteric NSCs led to significantly increased nNOS expression in the ENS, to preserved intestinal integrity with elongation of villous length, to significantly improved intestinal motility, and to significantly increased survival after experimental NEC. These results support a future potential clinical role for enteric NSC transplantation in patients with NEC.

## Abbreviations

chAT: Choline acetyl transferase; DMEM/F12: Dulbecco’s modified Eagle’s medium/Ham’s nutrient mixture F12; ENS: Enteric nervous system; GFAP: Glial fibrillary acid protein; GFP: Green fluorescent protein; i.p.: Intraperitoneal; LMMP: Longitudinal muscle–myenteric plexus; NEC: Necrotizing enterocolitis; nNOS: Neuronal nitric oxide synthase; NSC: Neural stem cell; RT-PCR: Reverse-transcription polymerase chain reaction.

## Competing interests

The authors declare that they have no competing interests.

## Authors’ contributions

YZ conceived of the study, carried out all experiments, acquired and interpreted the data, and drafted the manuscript. JY carried out the animal experiments and interpreted the data. DJW, LAB and MAM collected human intestinal specimens, and acquired and interpreted clinical data. YS performed the immunohistochemistry experiments, performed statistical analyses and interpreted the data. GEB conceived of the study, participated in its design and coordination, carried out critical revision of the manuscript, and obtained funding. All authors read and approved the final manuscript.
